# Vitamin D-Resistant Rickets and Cinacalcet—One More Favorable Experience

**DOI:** 10.3389/fped.2018.00376

**Published:** 2018-11-28

**Authors:** Ramona C. Nicolescu, Jacques Lombet, Etienne Cavalier

**Affiliations:** ^1^Division of Endocrinology and Diabetes, Department of Pediatrics, Centre Hospitalier Regional Citadelle, University of Liège, Liège, Belgium; ^2^Division of Nephrology, Department of Pediatrics, University Hospital Center of Liège, Liège, Belgium; ^3^Department of Clinical Chemistry, University Hospital Center of Liège, University of Liège, Liège, Belgium

**Keywords:** rickets, alopecia, vitamin D receptor, 1, 25-dihydroxyvitamin D_3_, cinacalcet

## Abstract

Hereditary vitamin D-resistant rickets (HVDRR) is an autosomal recessive disorder characterized by early onset of severe rickets, with a complete triad of clinical, biochemical and skeletal abnormalities. Homozygous or heterozygous mutations in the vitamin D receptor (*VDR*) gene leading to complete or partial target organ resistance to the action of 1α, 25-dihydroxyvitamin D3 (the active form of vitamin D) are responsible for HVDRR. Theoretically the therapeutic goal is to overcome this tissue resistance, and to normalize calcium and phosphate homeostasis. Practically, the treatment could be oriented to correct the secondary hyperparathyroidism to avoid long-term negative impact on bone health. The conventional therapeutic strategy (high-dose calcium plus active vitamin D metabolites) gives variable responses in magnitude and duration. We report a case of HVDRR with heterozygous mutation in the *VDR* gene, neonatal alopecia, and a severe clinical phenotype diagnosed at the age of 30 months who showed unsatisfactory response to traditional therapy. The short-term responsiveness to cinacalcet was encouraging, with adequate correction of phosphate-calcium homeostasis and significant improvement of clinical and radiological status at 6 months of treatment.

## Introduction

A 30-month-old female toddler was referred to our clinic with stunted growth and developmental delays (walking difficulties). Her antenatal course had been unremarkable. At birth, the baby had no scalp hair and her first dermatological evaluation led to the diagnosis of alopecia totalis, without identified etiology.

The toddler's medical history was unremarkable. She was on no medication and her immunization schedule was up to date. Her family history was negative for short stature or skeletal disorders. Her parents, coming from North Africa, are consanguineous and show normal phenotype.

The child was regularly followed by the general practitioner and after the age of 12 months, a progressive growth deceleration was noted. At age of 24 months, she was not yet walking and was referred for a neurology consultation. The review of systems was negative with no history of falls or other traumatic injuries.

On presentation in our clinic, the patient was alert, smiling and interactive, with normal vital signs. Her anthropometric parameters were weight 11 kg (15^th^P), length 80 cm (< 3^rd^P), and head circumference 51 cm (97^th^P). She did not have any midline abnormalities. She had alopecia, sparse eyebrows, and eyelashes without other skin or scalp lesions (papular lesions). The pulmonary, cardiovascular, and abdominal examinations were normal.

The musculoskeletal examination revealed frontal bossing, relatively large head, widening of wrists, prominent genu varum, and deformed lower limbs. Her neurological exam, with developmental milestones (language, fine motor, and social skills) was normal, except difficulty in walking (delay in motor maturation).

The first results of blood work indicated hypocalcemia (1.7 mmol/L), hypophosphatemia (1.1 mmol/L) with very high alkaline phosphatase (ALP) levels (2,380 U/L), and parathyroid hormone (PTH) titer (427 ng/mL). Her total 25-hydroxyvitamin D [25(OH) D_2_] level was low (13 ng/mL) and the 1,25-dihydroxyvitamin D3 [1,25(OH)_2_ D_3_] level was markedly elevated (528 pg/mL). Serum FGF-23 was undetectable. Her renal, liver and thyroid function tests were normal. Urine analysis showed a normal Ca/creatinine ratio (0.04), and a tubular threshold for phosphate [measured as tubular phosphate per liter glomerular filtration rate (TP/GFR)] at 1, indicating an appropriate-for-age renal phosphate handling (1) (Table 1).

**Table 1:** Patient's serum biochemistry evolution and treatment protocol.

	**Calcium**	**Phosphate**	PTH^*^	**ALP**	**25(OH)D_2_**	**1,25(OH)_2_D_3_**	**U Ca/cr (urine sample)**	**TmP/GFR**
**Range**	**2.2–2.54 mmol/L**	**1.41–2.17 mmol/L**	**15–65 ng/mL**	**151–342 U/L**	**> 30 ng/mL**	**20–80 pg/mL**	**<0.7 mmol/mmol**	**1.04–2.79mmol/L**
**Baseline**	**1.7**	**1.1**	**427**	**2,380**	**13**	**528**	**0**	**1.06**
**FIRST PHASE TREATMENT**								
5,000 U vitamin D_3_ 3,000 mg Ca/kg/d 1 month								
Start	1.7	1.1	427	2,380	13		0	1.06
End	1.69	1.09	574	2,132	37		0.19	1.43
**SECOND PHASE TREATMENT**								
iv calcium 45 mg/kg/d 4 months								
Start	2.23	0.63	427	1,653			0.78	1.43
End	2.18	0.98	350	1,569			0.94	1.47
**THIRD PHASE TREATMENT**								
Cinacalcet 0.27 mg/kg/d 2 months								
Start	2.18	0.98	350		62		2.57	1.47
End	2.4	1.91	94					2.85
6 months of follow-up	2.33	1.51	23	304				2.15

*PTH, parathyroid hormone*,

* *measured by an electrochemiluminescence assay that detects full-length PTH (1–84). ALP, alkaline phosphatase. 25 (OH)D_2_, 25-hydroxyvitamin D; 1,25(OH)_2_D_3_, 1,25-dihydroxyvitamin D3; U Ca/cr, urinary calcium/creatinine ratio; TmP/GFR, tubular maximum reabsorption of phosphate per unit of glomerular filtrate; iv, intravenous*.

**Figure 1:**
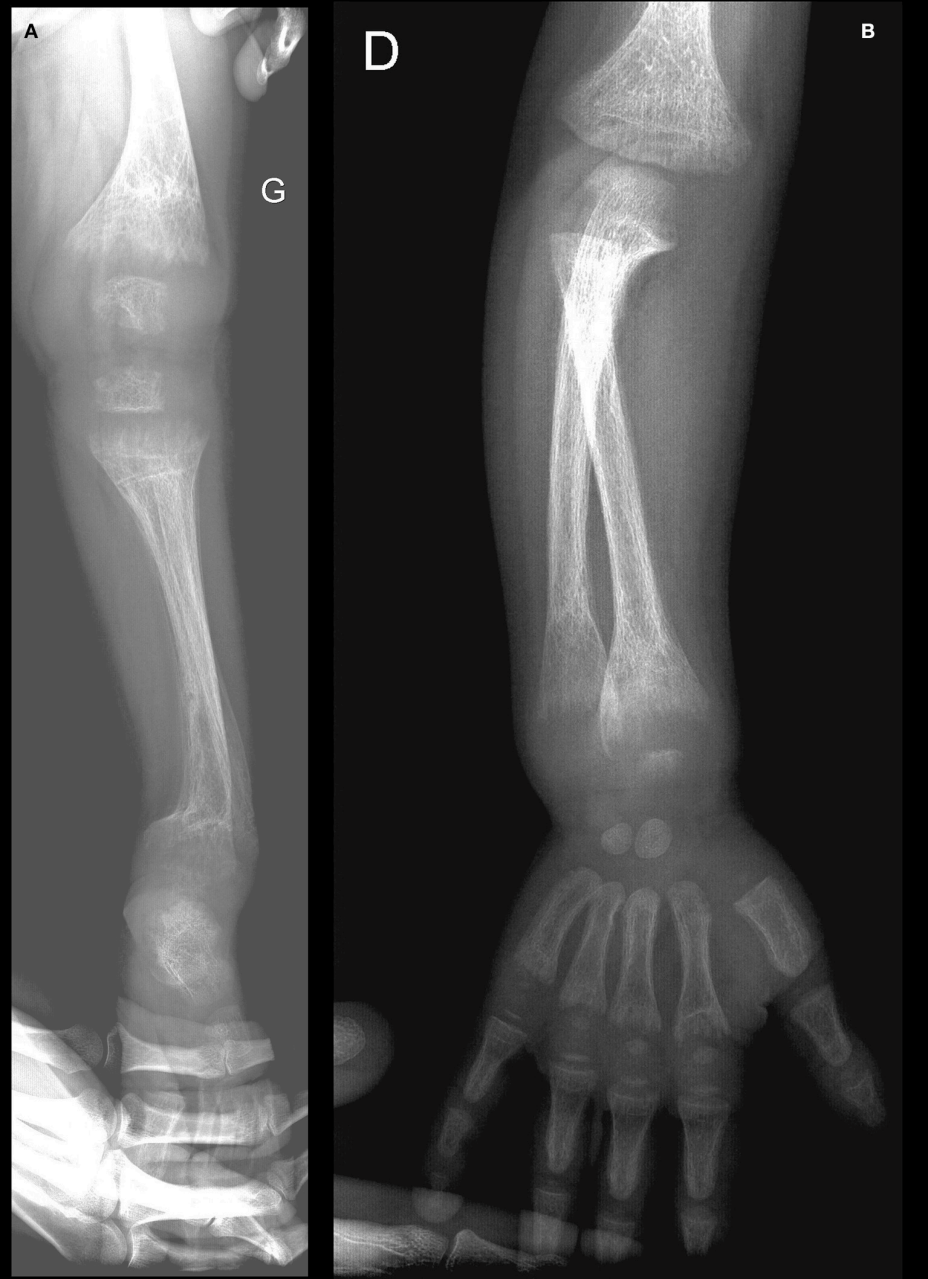
**(A,B)** Rachitic changes of the lower **(A)** and upper **(B)** extremity bones (at diagnosis).

The skeletal survey demonstrated abnormalities consistent with rickets, including cupping and fraying of the metaphyses of the long bones with widening of the growth plates, and generalized osteopenia (Figure 1). Some old and healed fractures (of the left tibia, left humerus, and both forearms) were also noted. The kidneys appeared to be normal on ultrasound.

## Background

Hereditary vitamin D-resistant rickets (HVDRR) is an autosomal recessive disease caused by abnormality of the vitamin D receptor (*VDR*) gene. The *VDR*, as mediator of the action of vitamin D, is composed of three distinct regions, and mutations in all its domains have been identified.

Mutations in the DNA-binding domain (DBD) prevent the *VDR* from binding to DNA, causing total 1,25(OH)_2_D_3_ resistance and a severe phenotype of rickets (2). Mutations in the ligand-binding domain (LBD) may disrupt *VDR* binding, or prevent co-activators from binding to the *VDR*, causing partial or total hormone resistance (2).

Patients with HVDRR have rickets that is not infrequently coupled to alopecia, so this ectodermal sign can raise clinical suspicion to differentiate vitamin D- resistant rickets type 1 (1 alpha hydroxylase deficiency) from type 2 (*VDR* gene mutations).

The biochemical presentation is similar to that of nutritional rickets, with severe disorder of calcium homeostasis: hypocalcemia that may be severe (leading to significant seizure activity), hypophosphatemia secondary to compensatory hyperparathyroidism, and elevated alkaline phosphatase activity. The level of 25(OH)D_2_ is usually normal, and the level of 1,25(OH)_2_D_3_ is very high, confirming the presence of vitamin D tissue resistance.

The confirmatory diagnosis is genetic, with more than 100 distinct mutations described today.

The treatment of HVDRR has not been standardized. Deficient calcium absorption from the gut with resulting secondary hyperparathyroidism is the main pathophysiological mechanism responsible for the skeletal abnormalities. Intravenous calcium therapy is the principal therapy employed to overcome calcium malabsorption in the gut, and has variable effects on restoration of normal calcium levels.

Using the model of secondary hyperparathyroidism occurring from other etiologies, a calcimimetic molecule, with inhibitory effect on parathyroid secretion could be used to reduce severe secondary hyperparathyroidism of HVDRR, and therefore to normalize serum phosphate, to heal rickets and to prevent worsening of it.

As reported in the literature, different *VDR* mutations are responsible for the HVDRR phenotype that includes almost the same clinical, biological, and radiological findings. A variety of pharmacological interventions were used over the time, with different outcomes. Table 2 gives a comparative summary of the recently reported case of HVDRR in toddlers.

**Table 2:** Characteristics and therapeutic response of the recently published cases of toddlers with HVDRR and *VDR* gene mutations.

**Genotype**	**Demographic data**	**Clinical data**	**Biochemical data**	**Drug(s) used**	**Treatment duration**	**Response to treatment**	**References**
Arg80Gln missense mutation in the *VDR* DNA-binding domain	30-month-old girl Consanguineous parents from North Africa Child's country of origin-Belgium	Total neonatal alopecia Stunted growth Developmental delay Upper and lower extremity rachitic deformities	Hypocalcemia Hypophosphatemia Very high ALP and PTH Low 25(OH)D_2_ Very high 1,25(OH)_2_ D_3_	Vitamin D + oral calcium iv calcium 45 mg/kg/d Cinacalcet 0.27 mg/kg/d	1 month 4 months 2 months	No improvement No improvement Clinical improvement with biological and radiological healing	Current case report
Homozygote stop-codon mutation (c.148 C >T) in exon 2 of the *VDR* gene Homozygote stop-codon mutation (c.148 C >T) in exon 2 of the *VDR* gene	2.5-year-old girl 4-month-old girl Consanguineous parents from Turkey Children's country of origin-Turkey	Alopecia Failure to thrive Seizures Lower extremity deformities Nearly total neonatal alopecia No clinical abnormalities	Hypocalcemia Hypophosphatemia Very high ALP and PTH Low 25(OH)D_2_ Normal 1,25(OH)_2_D_3_ Normal serum Ca and P levels at 4 months, but hypocalcemia, high PTH and ALP at 6 months	Oral elemental Ca 2–4 g/d + calcitriol 2–4 μg/d + phosphorus 1 g/d iv calcium 150 mg/kg/d Cinacalcet 0.25–0.40 mg/kg/d + oral calcium 4 g/d + calcitriol 6 μg/d Calcitriol 2–4 μg/d + intermittent oral and iv calcium Cinacalcet 0.25 mg/kg/d + oral elemental Ca 1 g/d + calcitriol 2 μg/d	14 weeks 4 months 5 months 2 months 5 months	No improvement Clinical and radiological improvement, but recurrence when therapy off Biological and radiological healing Temporary metabolic and radiological improvement Biological and radiological healing	(3)
Two compound heterozygous mutations in the ligand-binding site of *VDR* heterogeneous missense mutation of Arg274 (Arg274His) in exon 9 heterogeneous missense mutation of Arg73 (Arg73Glu) in exon 5	19-month-old female Non consanguineous parents of Hispanic origin Child's country of origin-Unites States of America	Poor growth	Hypocalcemia Normal phosphatemia Very high ALP and PTH Low 25(OH)D_2_ Very high 1,25(OH)_2_D_3_	Ergocalciferol 4,000 IU/d + oral and iv calcium Calcitriol 8 μg/d	2 months 6 months	Fluctuating calcemia Biological and radiological healing	(4)
Homozygous p.K45E mutation located in the DNA-binding domain of the *VDR* gene (5/8) Homozygous p.T415R mutation located in the ligand-binding domain in the *VDR* gene (1/8)	8 toddlers (age between 8 and 36 months) Consanguineous parents from Tunisia (7/8) Children's country of origin-Tunisia	Alopecia (7/8) Growth retardation (7/8) Skeletal abnormalities (7/8) Motor developmental delay (5/8)	Hypocalcemia (8/8) Hypophosphatemia (8/8) Very high ALP and PTH (8/8) High 1,25(OH)_2_D_3_ (8/8) Normal 25(OH)D_2_ (7/8)	Alfacalcidol 12 −20 μg/d + oral calcium 2 g/d (8/8) Alfacalcidol 6 μg/d + iv calcium + oral calcium 1 g/d (6/8)	6 months 2.6 years	No clinical and laboratory improvement Clinical and radiological improvement	(5)
Homozygous nonsense mutation p.Arg73-Ter, in the DNA-binding domain of the *VDR* gene and uniparental disomy of maternal chromosome 12	2-year-old girl Non-consanguineous parents Child's country of origin-Japan	Partial alopecia Short stature Gait instability Lower extremity deformities	Hypocalcemia Hypophosphatemia Very high ALP and PTH Normal 25(OH)D_2_ High 1,25(OH)_2_D_3_	Alfacalcidol 5 μg/d + oral calcium 60 mg/kg/d Oral calcium 250 mg/kg/d	12 months 20 months	No improvement Biological and radiological improvement	(6)
Homozygous splice acceptor mutation (c.147–2A>T) in intron 2 of the *VDR* gene	15-month-old boy Consanguineous parents from Pakistan Child's country of origin-United Kingdom	Alopecia Growth failure Gross motor developmental delay	Hypocalcemia Hypophosphatemia Very high ALP and PTH Low 25(OH)D_2_ Very high 1,25(OH)_2_D_3_	Ergocalciferol 6000 IU/d + elemental calcium po iv calcium	3 months 8 months	No improvement Biological and radiological healing	(7)
Missense mutation in exon 2 in the DNA-binding domain of *VDR*	13-month-old boy Child's country of origin-United States of America	Partial alopecia Failure to thrive Rachitic deformities	Hypocalcemia Hypophosphatemia Very high ALP and PTH Low 25(OH)D_2_ Very high 1,25(OH)_2_D_3_	Oral calcium + calcitriol iv calcium 450–600 mg/d Cinacalcet, 0.25–1 mg/kg/d	6 months 6 months 19 months	No improvement Biological and radiological improvement, but recurrence when therapy off Biological and radiological healing	(8)
Skipped exon 8 in the *VDR* ligand-binding domain	24-month-old girl Consanguineous parents from Mexico Child's country of origin-United States of America	Partial neonatal alopecia Multiple fractures Generalized hypotonia with motor delay Respiratory events with acute respiratory distress	Hypocalcemia Hypophosphatemia Very high ALP and PTH Low 25(OH)D_2_ Very high 1,25(OH)_2_D_3_	Ergocalciferol 4000 to 20,000 IU/d and then calcitriol 70–110 ng/kg/d + elemental calcium po + phosphorus iv calcium 30 to 147 mg/kg/d High-dose oral calcium	4.5 months	No improvement Normal calcemia and PTH Radiographic signs of healing	(9)

## Discussion

In the context of parents consanguinity, neonatal alopecia, early and severe rickets phenotype and high level of 1,25(OH)_2_D_3_, the suspicion of HVDRR was high. Direct sequencing of the genomic DNA revealed a single base substitution at position 80 (Arg80Gln) in exon 3 of the binding-domain of the *VDR* gene, and confirmed the diagnosis.

Several therapeutic interventions were initiated successively, designed to reduce the secondary hyperparathyroidism.

During the first phase of treatment the patient received oral calcium as calcium carbonate (3,000 mg daily) and high doses of vitamin D_3_ (5,000 IU/day). At the end of this 1-month treatment, 25(OH)D_2_ normalized but the serum phosphate decreased and the hypocalcemia persisted.

Classically, the second therapeutic step should include supra-physiological doses of oral calcium and calcitriol. We did not prescribe calcitriol for 2 reasons: theoretically, children with *VDR* defects and alopecia are unresponsive to oral calcium, vitamin D, or calcitriol (10, 11), and practically, in Belgium the drug is available only as capsules containing 0.25 or 0.50 μg (so very difficult to administer in a child of 30 months), and its high cost is not covered by the medical insurance.

In such circumstances, we decided to administer an intravenous (iv) infusion of 10% calcium chloride (up to 500 mg/day, 45 mg/kg/day) with close monitoring of blood and urine calcium levels. The iv calcium administration via a central line was continued for 4 months, with a mean infusion time of 16 h/day and no adverse reactions (infections). Stable, normal serum calcium levels occurred, with persistent high PTH and ALP levels and transient hypercalciuria (U Ca/cr in urine sample at 0.78 and 0.94) (Table 1) developed after 2 months. The renal ultrasound was normal, no nephrocalcinosis. Clinically, some improvement was noted, with better walking and reported relief of bone pain.

At this point (failure to suppress PTH secretion), we reviewed the international experience reported on cinacalcet use in children with secondary hyperparathyroidism and decided to use it in the third phase of treatment.

The treatment was started in the hospital, in the setting of normal serum calcium levels, at a dose of 0.27 mg/kg/day in once-daily oral administration. The iv calcium infusion remained unchanged. Close monitoring of serum and urine calcium levels was initiated (given the risk of hypocalcemia and hypercalciuria).

**Figure 2:**
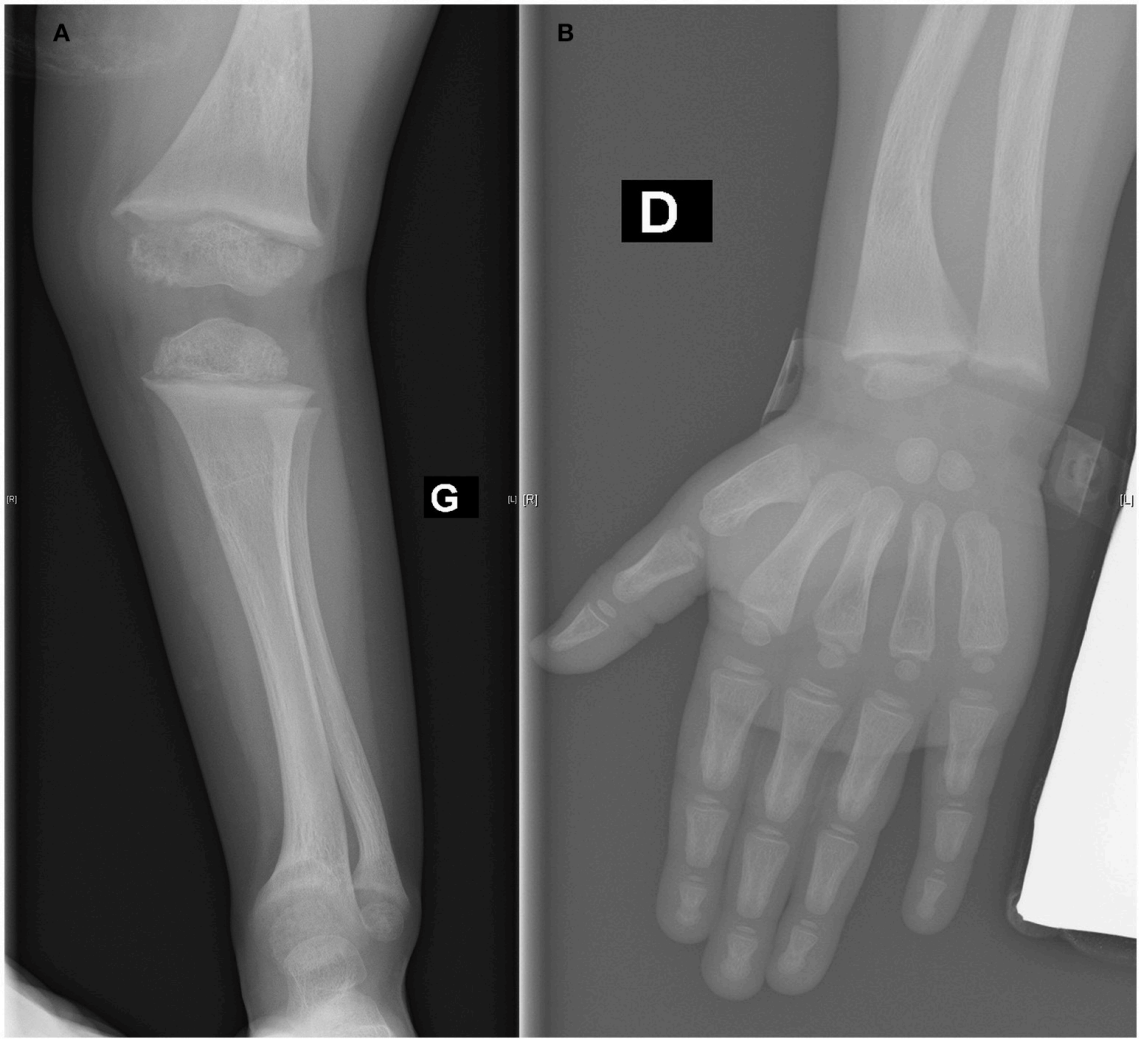
**(A,B)** Healing of rachitic lesions at the lower **(A)** and upper **(B)** extremity (12 months after diagnosis).

Cinacalcet was administered for 2 months, and the treatment normalized serum phosphate, PTH, and ALP levels. Hypocalcemia did not occur during treatment. At the completion of cinacalcet treatment and for 6 months afterward, hyperparathyroidism did not recur (with the PTH at 23 ng/mL) and the patient's calcium, phosphate, and ALP levels remained in the normal ranges (Table 1). Twelve months after the diagnosis, unsurprisingly, the child's height was 89 cm (3^rd^ P) (growth velocity of 9 cm/12 months) and she walked normally. The alopecia remained unchanged. The rachitic lesions on the X-ray images resolved (Figure 2). Currently, the child is being prescribed oral vitamin D_3_ 25,000 units/month and receives an intravenous calcium infusion (9 mg/kg/day) 3 times a week over 12 h/day and consumes a normal diet.

In this case, the primary presenting sign was alopecia totalis with sparse eyebrows that passed undiagnosed even when growth failure and gross motor developmental delay completed the clinical picture.

Early in the evolution, isolated alopecia is not very helpful to point to the right diagnosis, but the possibility of HVDRR should be on the differential diagnosis, and a regular clinical and biological assessment protocol could be initiated.

This patient's genotype was previously described and is known to be associated with a near total resistance to 1,25(OH)_2_D_3_ and alopecia (2). Alopecia can have variable presentations, and its pathogenesis and correlation with the patient's genetic background remain partially explained.

The HVDRR treatment should target the main biological abnormality, the exaggerated and sometimes uncontrolled PTH secretion that has severe impact on bone and usually a stepwise approach is recommended.

Vitamin D functions to maintain plasma calcium homeostasis through the action of plasma 1,25(OH)_2_D_3_ and VDR on kidney, small intestine and bone.

Calcium is absorbed in the gut by both an active transcellular pathway located largely in the duodenum and upper jejunum, and by a passive paracellular pathway through tight junctions in the entire gut. 1,25(OH)_2_D_3_ is the major stimulator of active intestinal calcium absorption, and can also enhance paracellular calcium diffusion. In HVDRR patients, because of a lack of VDR signaling in the intestine, calcium absorption capacity is both limited and highly variable and hypocalcemia, secondary hyperparathyroidism, and hypophosphatemia develop. The efficacy of vitamin D_3_ and high doses of oral calcium therapy is low, even physiologically the high doses of oral calcium could use the vitamin D-independent, paracellular pathway of intestinal absorption. Simultaneously, excessive oral calcium administration could induce gut sequestration of dietary phosphate, aggravating the initial hypophosphatemia.

High doses of iv calcium, able to bypass the abnormal intestinal absorption (12) and calcitriol can have variable responses (incomplete or unsustainable corrections of secondary hyperparathyroidism with temporary metabolic improvement) (3, 4). Two possible mechanisms could be discussed: (a) a certain variability of serum calcium levels between iv calcium infusions, with transitory normocalcemia and hypocalcemia, and subsequently, no consistent effect on PTH secretion and (b) a certain level of parathyroid tissue hyperplasia in the context of long-lasting hypocalcemia.

HVDRR-associated hypophosphatemia is mainly due to secondary hyperparathyroidism. Does 1,25(OH)_2_D_3_ tissue resistance imply also a variable intestinal phosphate absorption, responsible for hypophosphatemia? Currently, experimental evidence supports a bimodal pathway involved in intestinal phosphate absorption: a transcellular transporter-dependent pathway and a poorly defined paracellular route. The transcellular pathway involves the sodium-driven phosphate transporter NaPi-IIb and its function is regulated by dietary phosphate intake and 1,25(OH)_2_D_3_ (13). Specific removal of the Na^+^/Pi cotransporter NaPi-IIb from the mice intestinal epithelia results in almost complete loss of active ileal transport of phosphate leading to increased fecal excretion and a compensatory reduction of the renal output of phosphate (14).

The therapeutic target of HVDRR management is the hyperparathyroidism control. Cinacalcet, an allosteric modulator of calcium-sensing receptor (CaSR), with inhibitory effects on PTH secretion was used in pediatric patients and showed its efficacy to diminish exaggerated PTH secretion secondary to different etiological conditions (15–20). More specifically, when used as adjunctive therapy in HVDRR patients, cinacalcet restored their calcium and phosphate homeostasis, with normal PTH levels occurring over extended follow-up periods (3, 8).

In our patient, a 2-month-treatment with cinacalcet effectively controlled hyperparathyroidism, with complete restoration of calcium and phosphate homeostasis and healing of rickets.

## Concluding remarks

We described a case of HVDRR with neonatal alopecia and a favorable clinical, biological and radiological response to cinacalcet treatment. No side effects were reported, but caution is highly recommended because this drug can aggravate hypocalcemia and induce hypercalciuria

Our patient's history suggests attention to early diagnosis of HVDRR presenting as isolated alopecia and joins other case-reports on the efficacy and safety of cinacalcet in this entity.

Our results suggest that the phenotype-genotype correlation (alopecia and mutation in the *VDR* gene) may allow us to efficaciously orient the therapeutic approach. Calcium, vitamin D, calcitriol remain the first therapeutic choice (while waiting for genetic confirmatory results), but if these drugs cannot adequately improve biochemical abnormalities, an earlier use of a calcimimetic molecule to control PTH secretion should be considered.

A prospective multicenter study aiming to compare cinacalcet vs. standard treatment (high doses of oral and iv calcium and vitamin D) regarding long-term metabolic and radiological rachitic healing could potentially improve our management of HVDRR cases.

## Ethics statement

This case report was approved by the Ethics Committee of the Centre Hospitalier Regional de la Citadelle. Written informed consent was obtained from the patient's parent prior to presenting the case.

## Author contributions

JL has directly cared for the patient. RN and JL contributed to the analysis and interpretation of the data, participated actively in preparing the manuscript and approved the submitted final version. EC is acknowledged for his laboratory work and critical reading of the manuscript.

### Conflict of interest statement

The authors declare that the research was conducted in the absence of any commercial or financial relationships that could be construed as a potential conflict of interest.
